# Energetics of vacancy segregation to [100] symmetric tilt grain boundaries in bcc tungsten

**DOI:** 10.1038/srep36955

**Published:** 2016-11-22

**Authors:** Nanjun Chen, Liang-Liang Niu, Ying Zhang, Xiaolin Shu, Hong-Bo Zhou, Shuo Jin, Guang Ran, Guang-Hong Lu, Fei Gao

**Affiliations:** 1 College of Energy, Xiamen University, Xiamen City, Fujian Province, 361102, China; 2 Department of Nuclear Engineering and Radiological Science, University of Michigan, Ann Arbor, MI 48109 USA; 3 Department of Physics, Beihang University, Beijing 100191, China

## Abstract

The harsh irradiation environment poses serious threat to the structural integrity of leading candidate for plasma-facing materials, tungsten (W), in future nuclear fusion reactors. It is thus essential to understand the radiation-induced segregation of native defects and impurities to defect sinks, such as grain boundaries (GBs), by quantifying the segregation energetics. In this work, molecular statics simulations of a range of equilibrium and metastable [100] symmetric tilt GBs are carried out to explore the energetics of vacancy segregation. We show that the low-angle GBs have larger absorption length scales over their high-angle counterparts. Vacancy sites that are energetically unfavorable for segregation are found in all GBs. The magnitudes of minimum segregation energies for the equilibrium GBs vary from −2.61 eV to −0.76 eV depending on the GB character, while those for the metastable GB states tend to be much lower. The significance of vacancy delocalization in decreasing the vacancy segregation energies and facilitating GB migration has been discussed. Metrics such as GB energy and local stress are used to interpret the simulation results, and correlations between them have been established. This study contributes to the possible application of polycrystalline W under irradiation in advanced nuclear fusion reactors.

Point defects are the simplest defects of materials. In non-equilibrium processes such as displacement cascades^1^,^2^,^3^,^4^, an excess of point defects can be created. The subsequent evolution of these point defects may lead to the formation of dislocation loops^1^,^2^,^5^ and voids^6^,^7^,^8^, which inevitably induces the degradation of mechanical properties, such as swelling, hardening, embrittlement and even direct failure. As an intrinsic two-dimensional defect in polycrystalline materials, grain boundary (GB), where grains with different crystallographic orientations join, has tremendous influence on the thermal, electrical and mechanical properties of materials. It has long been established that GBs can act as efficient sources or sinks for point defects^2^,^9^,^10^,^11^,^12^,^13^,^14^,^15^. This effect of absorbing or emitting point defects can have significant implications for nanomaterials with a high volume fraction of GBs or interfaces. Prior experimental studies^16^,^17^,^18^ and atomistic simulations^10^,^11^,^17^ revealed that nanomaterials generally show better radiation resistance in comparison with conventional coarse-grained materials. The underlying mechanism has been revealed by Bai, Uberuaga and coworkers^10^,^19^, demonstrating that interstitial-loaded GBs, acting as a source, can emit interstitials to annihilate vacancies present in the near-GB region.

A quantitative picture of defect segregation energetics is essential to the understanding of dynamic processes of defect evolution in materials under irradiation. On this very topic, density functional theory calculations^20^,^21^ and atomistic simulations^9^,^10^,^11^,^22^,^23^,^24^,^25^,^26^,^27^,^28^,^29^ have been frequently used to describe the segregation energetics of point defects (including impurities) to a series of GBs. For example, Tschopp, Solanki and coworkers have performed extensive atomistic simulations to investigate the effects of GB character on the formation energetics of point defects, such as vacancy^9^,^22^,^23^,^24^,^30^, self-interstitial^9^, hydrogen^25^,^26^, helium^26^,^27^, carbon^26^,^28^ and various other impurities^23^,^26^. Aiming to mitigate radiation damage, Uberuaga, Demkowicz and coworkers have conducted massive multiscale simulations^19^,^31^,^32^,^33^,^34^,^35^,^36^,^37^,^38^ to understand the effects of interface structure on point defect segregation, defect mobility, defect recombination and GB sink efficiency. Jiang *et al*.^29^ demonstrated that GB types can make a difference in determining the sink strength and radiation tolerance of GBs. Another important aspect of this phenomenon is that point defect segregation can lead to GB or interface structural transformation^39^,^40^,^41^,^42^, which further influences GB migration behavior^43^,^44^ and sink strength^45^.

Tungsten (W) is considered as a leading candidate for plasma-facing materials in future nuclear fusion reactors. The bombardment of high-energy neutrons generated in deuterium-tritium nuclear reaction can be devastating. Therefore, it is of great interest to investigate the point defect segregation energetics in bcc W. Though efforts^46^ have been made to understand this issue, complete data set regarding the influence of GB character on point defect segregation energetics is still lacking. Furthermore, the nature of GB vacancies along with its potential influence on GB migration, and the effect of different GB states, corresponding to each particular misorientation angle, on vacancy segregation have been unclear. The objective of this work is to gain further understanding on these key issues based on atomistic simulations using an embedded atom method (EAM) potential, and possibly provide inputs of energetics and vacancy absorption length scales for modelling and simulation of W at larger scales.

## Results

### Grain boundary character and local hydrostatic stress

**Figure 1:**
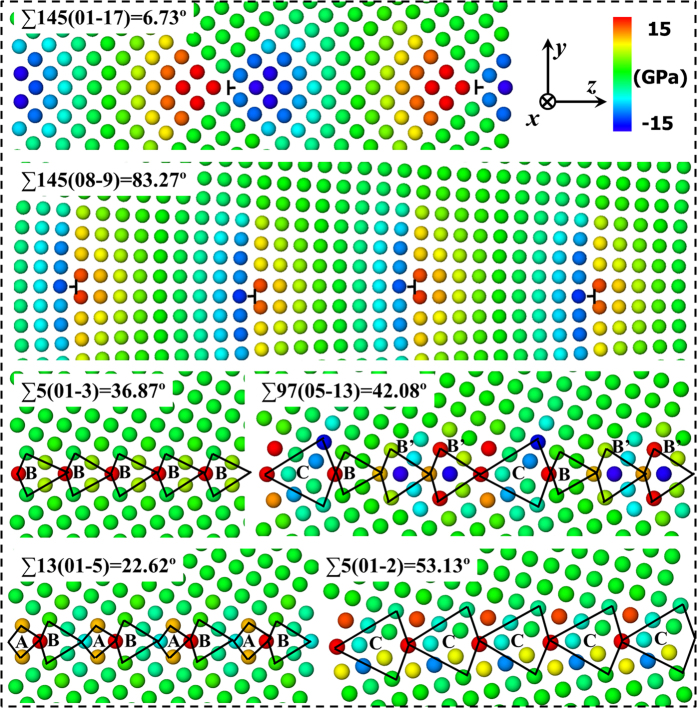
Grain boundary structures and local hydrostatic stress for selected [100] STGBs. The labelled structural units are consistent with previous works on bcc iron^9^,^22^. Note that ∑5(01-3) and ∑5(01-2) are favored boundaries as they (contain the basic structural units B and C, respectively) along with the structural units of their variants and the units of the perfect lattice constitute the other high angle STGBs. The low-angle STGBs can be readily described by previously proposed dislocation models^48^,^49^. Different numbers of coincidence site lattice (CSL) periodicities are depicted to facilitate easy comparison. The range of −15 to 15 GPa is used for visual clarity. Here, the upper grain is taken as the reference lattice for all crystallographic indices with *x* being the [100] tilt axis and *y* the GB normal.

Grain boundary character described in terms of GB energy and structure has long been studied in bcc, fcc and hcp metals^9^,^47^,^48^,^49^,^50^,^51^. The 36 [100] equilibrium symmetric tilt grain boundaries (STGBs) with misorientation angle *θ* varying between 5.5° and 84.5° have been previously explored in our most recent work regarding shear-coupled GB migration^52^. The extra energies of these equilibrium STGBs are presented in Supplementary Table 1. Typical GB structures which possess all the features of [100] GB family are illustrated in Fig. 1. Specifically, the lower end of the low-angle GBs (≤15°) classified as low angle-I present a discrete array of 〈100〉 dislocations, while the higher end of the low-angle GBs (≥75°) classified as low angle-II consist of an array of resolved 1/2 〈111〉 dislocations^53^. According to the previous dislocation models, these two kind of low-angle GBs can be satisfactorily described by the type 2 tilt wall model^48^,^49^ and type 1 tilt wall model^48^,^49^, respectively.

The structures of the high-angle STGBs can be readily characterized by the well-established structural unit model^9^,^47^,^54^, i.e., the STGBs can be determined by a combination of structural units from the favoured ∑5(01-3) (consists of B units) and ∑5(01-2) (consists of C units) boundaries, variant of the basic structural unit such as B′ and those from the perfect lattice A and A′, as shown in Fig. 1. For instance, ∑13(01-5) and ∑97(05-13) can be expressed as ∑13(01-5) = 2 A + 2B and ∑97(05-13) = 2B + 4B′ + 2 C, respectively. Another important feature of the STGBs is that they are not necessarily perfectly mirror-symmetric due to the in-plane translations, as clearly shown by the low-angle ∑145(08-9) or the high-angle ∑5(01-2) GBs. This kind of broken symmetry has also been found in several previous literatures^55^,^56^. Regarding the stress fields of the GBs, it has been recognized that GBs do not produce long-range stress fields. Figure 1 shows that the local hydrostatic stress concentrates in the GB region and its site to site values can vary drastically from −30 to 30 GPa for all the [100] STGBs studied here, which is important in determining the vacancy segregation energetics.

### Profiles of vacancy segregation energetics

**Figure 2:**
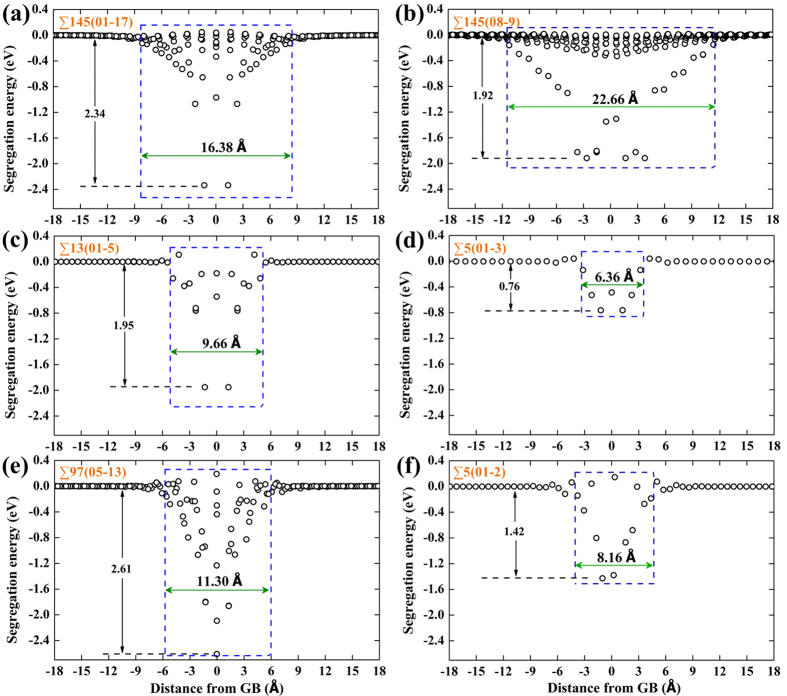
Vacancy segregation energy as a function of distance from the selected GBs corresponding to Fig. 1. The blue dashed box is defined as the GB absorption region where there are sites having segregation energies of −0.12 eV or lower at any distance within this region. The reference vacancy formation energy in a perfect bulk lattice is 3.631 eV.

**Figure 3:**
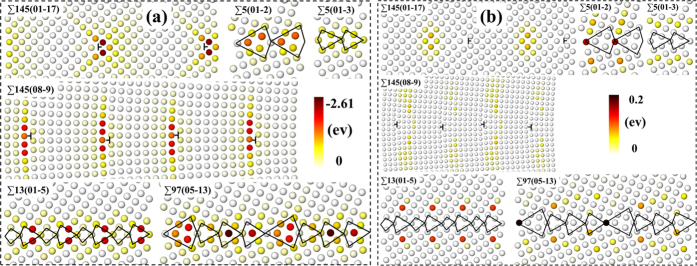
Vacancy segregation energy as a function of spatial position projected onto the (100) plane. (**a**) Attractive sites; (**b**) repulsive sites. Noticeably, values of vacancy repulsion are about an order of magnitude lower than those of vacancy attraction.

The variations of segregation energy in the vicinity of GBs have been quantified by plotting the correlation between vacancy segregation energy and the distance from the GB, which can be further complemented by drawing the spatial distribution map of the segregation energetics. Figure 2 shows the vacancy segregation energy as a function of distance for the STGBs depicted in Fig. 1. The dependence of vacancy segregation energy on spatial positions is shown in Fig. 3. The segregation energy is closely related to the vacancy sites with respect to GB dislocation cores or structural units. A comparison between Figs 1 and 3a suggests that the strongest vacancy attraction tends to occur at the compressive region. Quantitatively, the minimum segregation energies for these GBs vary significantly from −2.61 to −0.76 eV depending on the GB character. Note that slight repulsion of no more than 0.25 eV between the vacancy and the GB (see Fig. 3b) has been found in all the GBs, indicating that there are some unfavourable GB sites for vacancy segregation. This is consistent with recent atomistic simulations^9^ of vacancy-GB interaction in bcc iron using an EAM potential.

**Figure 4:**
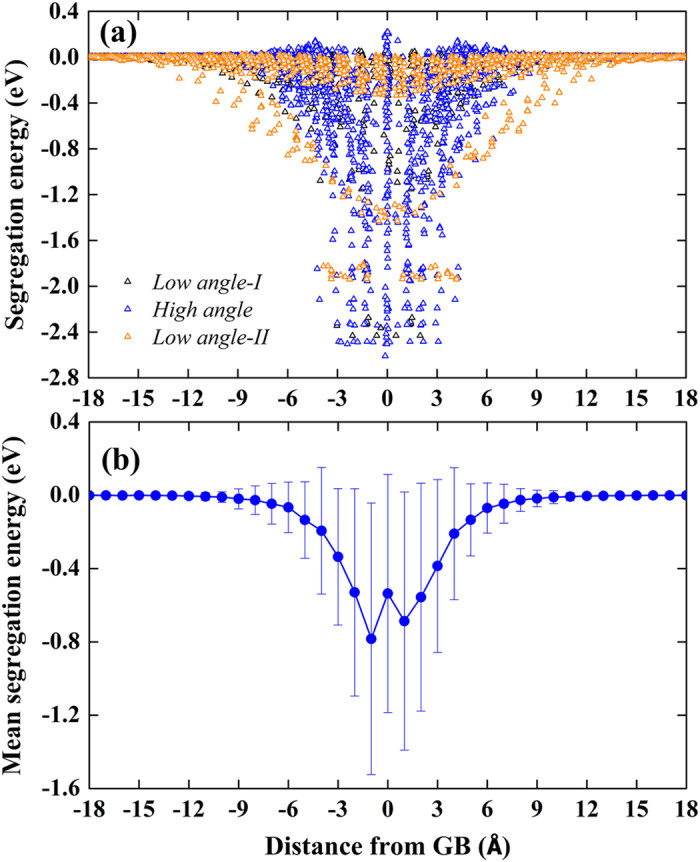
(**a**) Vacancy segregation energy and (**b**) mean vacancy segregation energy as a function of distance from the GB for all the [100] STGBs. For (**b**), vacancy segregation energies for sites in each 1 Ǻ bin on both sides of the STGBs are averaged. The error bars present the standard deviation from the mean values.

To provide further insight, we plot in Fig. 4a the vacancy segregation energy as a function of distance from the GB for all the STGBs. It can be seen that there exists a correlation between GB type and vacancy absorption length scale. Low angle-II STGBs show distinctively larger absorption length than high-angle STGBs, whereas low angle-I STGBs have slightly larger absorption length scale than most of the high-angle STGBs, as also demonstrated in Supplementary Table 1. The mean segregation energy is commonly used to characterize GB sink strength^9^,^22^. Figure 4b shows the mean vacancy segregation energy as a function of distance from the GB. A striking feature is the remarkable deviations from the mean values as the vacancy approaches the GB plane, indicating the significant segregation energy site to site variations influenced by the complex local structural compositions and stress environment. The fact that the plot is slight off-symmetry confirms the broken mirror-symmetry for several STGBs illustrated above. Another interesting point is that the vacancies locating right at the GB plane, in many circumstances (see ∑145(01-17) and ∑13(01-5) in Fig. 3), are less energetically favourable than vacancy sites one atomic layer from the GB interface, consistent with prior works^9^,^22^.

**Figure 5:**
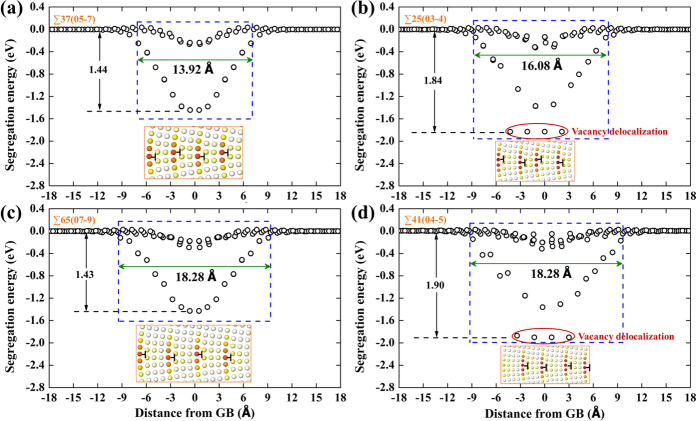
Vacancy segregation energy as a function of distance from (**a**) ∑37(05-7), (**b**) ∑25(03-4), (**c**) ∑65(07-9), and (**d**) ∑41(04-5) neighboring GBs. The blue dashed box is defined as the GB absorption region where there are sites having segregation energies of −0.12 eV or lower at any distance within this region. The insets are the corresponding GB structures colored according to their potential energies. Vacancies created in (**a,c**) remain compact, while delocalized vacancies are found in (**b and d**), which is reflected by the much lower minimum vacancy segregation energies. The reference vacancy formation energy in a perfect bulk lattice is 3.631 eV.

It is worth noting that vacancy delocalization has been commonly observed in most of the GBs, corresponding, in most cases, to sites having low segregation energies. The definition of “vacancy delocalization” is that the creation of the vacancy leads to marked relaxation further than the nearest neighbours^38^,^46^,^57^,^58^,^59^. This phenomenon is closely associated with the GB dislocation cores where the local compressive stress is the strongest. Structural visualization shows that, in low-angle GBs, vacancies in the bulk-like region between the dislocation cores remain compact, while they tend to delocalize as they approach the compressive side of the core. Since GB dislocation cores overlap in high-angle GBs, we observe, as expected, a high density of vacancy sites being delocalized. Figure 5 presents the vacancy segregation energetics of four neighboring GBs with similar character. By comparing Fig. 5a,c (with vacancy delocalization) with Fig. 5b,d (without vacancy delocalization), we show that vacancy delocalization leads to distinctively lower segregation energies, as can be seen from the large segregation energy gaps. It is sometimes, however, difficult to distinguish and quantify the boundaries between vacancies being delocalized and those that are not. The incompact nature of the vacancy structure can be important in several aspects. Kinetically, the migration mechanisms of the delocalized vacancy are dislocation-based, involving the nucleation of thermal kink pairs and multiple metastable states^38^,^57^. Since these states are separated by small barriers, the migration of delocalized vacancies is much easier than conventional vacancy migration with a single large activation energy barrier. This effect may have dramatic implications for GB mobility due to that the mobility of GBs have been revealed to be often dictated by the mobility of GB defects and impurities^33^,^60^. Our preliminary results show that well-defined vacancies inhibit GB migration (Supplementary Fig. S1) at low temperatures due to their lower mobility while delocalized vacancies facilitate GB migration (Supplementary Fig. S2).

### Dependence of vacancy segregation energetics on GB energy and local stress

**Figure 6:**
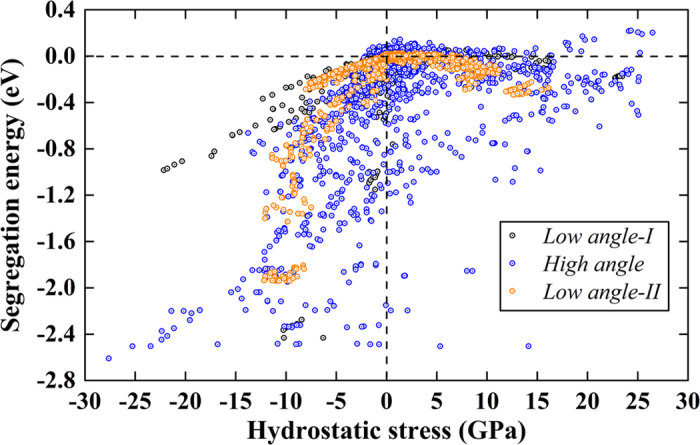
Dependence of vacancy segregation energy on local hydrostatic stress for all the [100] STGBs. The dashed lines are guide for the eyes. The first-order size effect and the second-order inhomogeneity effect dominate at the compressive and tensile region respectively.

The energetics of vacancy segregation is determined by the local coordination environment and the corresponding local stress fields. In this section, we will probe how local stress and GB energy influence the evolution of vacancy segregation energetics, and whether correlations between them can be established. Figure 6 shows the vacancy segregation energy as a function of the local hydrostatic stress for all the [100] STGBs. The effect of six independent local stress components on vacancy segregation is shown in Supplementary Fig. S3. Note that a quantitative description of vacancy-GB interaction using elasticity theory is not feasible, especially for high-angle GBs, due to the short-range stress field of the GBs. However, a qualitative interpretation can be given. According to elasticity theory, the vacancy-GB interaction comes from two main terms, i.e., the first-order size effect^61^,^62^ and the second-order inhomogeneity effect^61^,^62^. The first term only involves the hydrostatic field of the GB and vacancy relaxation volume, whereas the second term originates from the fact that vacancy having different elastic constants from the matrix will bring an inhomogeneity interaction, which comprises contributions from the hydrostatic and deviatoric parts of the stress tensor. The effective elastic constants of vacancy is essentially zero, hence this term only induces attraction. If we only consider the first-order size effect, the vacancy-GB interaction will be increasingly repulsive with increasing tensile stress, which completely deviates from the atomistic results here. Therefore, while the first-order size effect dominates the compressive region, the second-order inhomogeneity interaction term should be dominant at the tensile region possibly due to the small vacancy relaxation volume at this region.

**Figure 7:**
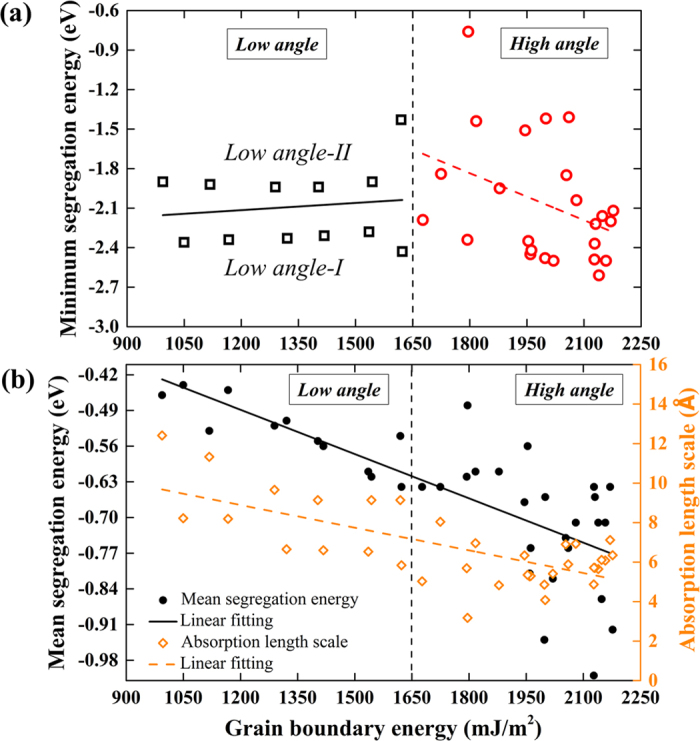
Dependence of (**a**) minimum vacancy segregation energy and (**b**) mean vacancy segregation energy and absorption length scale on GB energy for all the [100] STGBs. For (**a**), the linear correlation coefficients for the low- and high-angle GBs are 0.14 and −0.38, respectively. For (**b**), the linear correlation coefficients for the mean segregation energy and absorption length scale were calculated as −0.75 and −0.70, respectively, suggesting they are highly negatively correlated with the GB energy. Note that the vacancy length scale is defined as half of the values defined in Fig. 2. When calculating the mean segregation energy, only vacancies having segregation energies of −0.12 eV or lower are included.

Figure 7a shows the minimum vacancy segregation energy as a function of GB energy for all the [100] STGBs. The linear correlation coefficient has been used to quantify the magnitude of linear correlation. Here, values of −1 and 1 represent perfect negative and positive linear correlations, respectively. For the low-angle GBs, low angle-I and low angle-II GBs distribute at separate sides of the fitting line with an energy gap of ~0.5 eV, and the linear correlation coefficient of 0.14 indicates that the minimum segregation energy is, to a great extent, independent of the GB energy. In contrast, both the lowest value of −0.76 eV and the highest value of −2.61 eV are found in the high-angle GB category. The obtained linear correlation coefficient of −0.38 indicates a weak negative correlation. We further show in Fig. 7b the dependence of mean vacancy segregation energy (ranges from −0.44 to −1.01 eV) and absorption length scale (ranges from 3.18 to 12.41 Å) on GB energy for all STGBs. The corresponding linear correlation coefficients of −0.75 and −0.70 suggest reasonably strong negative correlations. Specifically, increasing GB energy, which involves a transition from the low-angle GBs to the high-angle ones, generally enhance vacancy segregation to the GBs, and, on the other hand, decreases the vacancy absorption length scale.

### Effect of GB metastability on vacancy segregation energetics

The understanding of GB equilibrium states is, undoubtedly, essential to the construction of the relationship between the GB structure and properties. Nevertheless, GBs in polycrystalline materials are more often found at nonequilibrium states under many circumstances, such as plastic deformation and radiation damage, and their multiplicity has been demonstrated by a wide range of experimental^63^,^64^,^65^,^66^,^67^ and computational studies^39^,^40^,^66^,^68^,^69^,^70^,^71^. In this section, we illustrate the effect of GB metastability on vacancy segregation energetics using four states of a typical long-period ∑89(03-13) GB.

**Figure 8:**
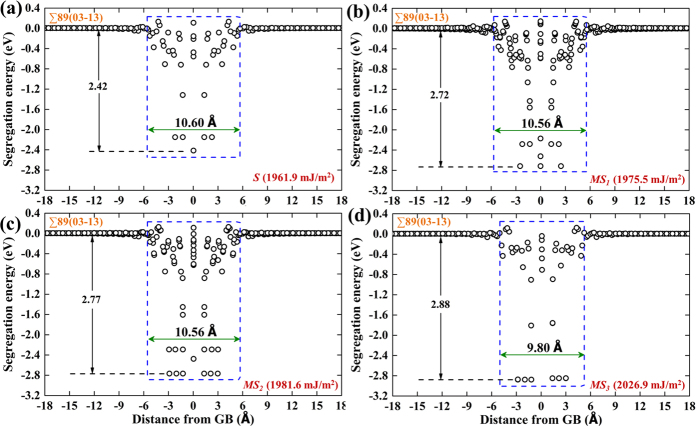
Vacancy segregation energy as a function of distance from the selected ∑89(03-13) GB states corresponding to (**a**) the most stable (denoted as ‘*S*’) and three metastable states (**b**) ‘*MS*_*1*_’, (**c**) ‘*MS*_*2*_’, and (**d**) ‘*MS*_*3*_’. The corresponding GB energies are given in the brackets at the lower right corner. The blue dashed box is defined as the GB absorption region where there are sites having segregation energies of −0.12 eV or lower at any distance within this region. The reference vacancy formation energy in a perfect bulk lattice is 3.631 eV.

We plot in Fig. 8, similar to Fig. 2, the vacancy segregation energy as a function of distance for one equilibrium (most stable, denoted as ‘*S*’) and three nonequilibrium (metastable, denoted as ‘*MS*’) STGBs. With increasing GB energies from 1961.9, 1975.5, 1981.6 to 2026.9 mJ/m^2^, we obtain correspondingly decreasing minimum segregation energies from −2.42, −2.72, −2.77 to −2.88 eV, and decreasing mean segregation energies from −0.76, −0.83, −0.88 to −0.93 eV. The vacancy absorption length scales have also been slightly decreased. The specific correlations are shown in Supplementary Fig. S4. These results support the negative correlations we established in Fig. 7 using equilibrium GBs of different misorientation angles.

**Figure 9:**
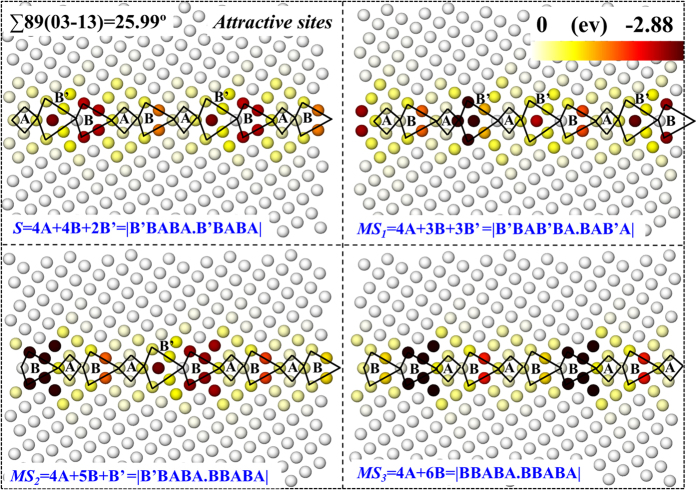
Vacancy segregation energy as a function of spatial position projected onto the (100) plane for ∑89(03-13) of different GB states (see the caption of Fig. 8 for more details). Only attractive sites are shown. These GB states can be, as we have demonstrated in the first section of “Results”, presented as different combinations of basic structural units A, B, and B′.

The corresponding vacancy segregation maps are depicted in Fig. 9 (the repulsive sites are shown in Supplementary Fig. S5). As we have discussed in the first section of “Results”, the four GB states can also be presented as different combinations of basic GB structural units A, B, and B′: *S* = 4 A + 4B + 2B′ = |B′BABA.B′BABA|, *MS*_*1*_ = 4 A + 3B + 3B′ = |B′BAB′BA.BAB′A|, *MS*_*2*_ = 4 A + 5B + B′ = |B′BABA.BBABA|, and *MS*_*3*_ = 4 A + 6B = |BBABA.BBABA|. Interestingly, the GB states *S, MS2* and *MS3* follow a similar trend in terms of the arrangement of B (B′) and A. The GB energies can be continuously lowered from *MS3, MS2* to *S* as B units are turned into B′. However, when we replace another B unit in *S* state to B′, shifting the GB to another metastable state *MS4* = 4 A + 3B + 3B′ = |B′BAB′A.B′BABA|, the GB energy increases to 1978.6 mJ/m^2^, larger in energy than *MS1* despite that they have the same combination of structural units. This indicates that GB energy is affected by both the combination and the permutation of GB structural units. At each GB state, neighboring vacancy sites having similar deep colour are found to be delocalized. Removing any one of them yields the same final defect structure, as can also be reflected by Fig. 8, in which sites of different distances from the GB plane have exactly the same level of segregation energies. The distinct feature in Supplementary Fig. S5 is that repulsive sites are only found a few atomic layers from the GB plane in state *MS*_*3*_, which is free of B′ unit, similar to those of ∑13(01-5) and ∑5(01-3) GBs presented in Fig. 3b.

## Discussion

In this section, the implications of this study will be presented. We have, unfortunately, not found any experimental studies on [100] W bicrystals regarding their energy and structure, comparisons will thus only be made against existing *ab initio* and atomistic simulation results. It is well documented that *ab initio* methods can describe elemental properties and interactions more accurately in comparison with classical atomistic simulations and one of the fundamental issues of the classical atomistic simulations is whether the interatomic potentials used is reliable and trustworthy^72^. Prior *ab initio* study by Mrovec *et al*.^73^ reported the GB energy of ∑5(01-2) as 2781 mJ/m^2^, and those of ∑5(01-3) as 2335 mJ/m^2^ and 2235 mJ/m^2^ for the mirror-symmetric and the displaced structures, respectively. The present EAM potential yields GB energies for the equilibrium states of ∑5(01-2) and ∑5(01-3) as 2000 mJ/m^2^ and 1796 mJ/m^2^, underestimating the GB energies by 781 mJ/m^2^ and 239 mJ/m^2^, respectively.

However, since GB structures have been frequently found to be with a broken mirror-symmetry and different GB site densities by both experimental^63^,^64^,^65^,^66^,^67^ and computational^39^,^40^,^66^,^68^,^69^,^70^,^71^ studies, it is important to recognize that the *ab initio* studies might also overestimate the GB energies due to their inherent inefficiency in thoroughly searching the GB phase space for the equilibrium states as that did by the classical simulations, in which thousands of GB in-plane displacements, atom eliminations, and sometimes high-temperature annealing are implemented^45^,^55^,^56^. Moreover, the high computational cost of *ab initio* calculations confines its usage to short-period GBs, which makes the comparison of the entire picture of GB structure (energy) versus misorientation angle unfeasible. Notably, the qualitative correlation between GB energy and misorientation angle (see Supplementary Fig. S6) agrees well with atomistic simulations of [100] STGBs in bcc iron^9^, tantalum^51^ and molybdenum^74^.

Regarding vacancy formation, the obtained bulk vacancy formation energy of 3.631 eV (see Supplementary Fig. S7) is in good agreement with experimental and *ab initio* values^75^. The *ab initio* calculations in our group recently obtained a minimum vacancy segregation energy of −1.53 eV for ∑5(01-3) GB, which doubles that (−0.76 eV) in the present work. However, the fact that a higher energy mirror-symmetric GB state, instead of the equilibrium displaced state, was used in the *ab initio* study support our conclusion in the previous section “Effect of GB metastability on vacancy segregation energetics”: GB states of higher energies lower the vacancy segregation energies. Quantitatively speaking, a GB energy increase of 13.6 mJ/m^2^ from *S* to *MS*_*1*_ state lowers the minimum vacancy segregation energy by 0.30 eV (Fig. 8 and Supplementary Fig. S4). We thus expect much larger decrease of vacancy segregation energy considering the energy difference^73^ of 100 mJ/m^2^ between the equilibrium displaced state and metastable mirror-symmetric GB state of ∑5(01-3) GB.

The bulk vacancy formation energy of 3.631 eV also indicates that the vacancy formation energies in all the equilibrium and metastable GB states considered in this work are positive, which evidences that the GBs we adopted are at low-energy states. It is, nonetheless, debatable whether researchers should pay primary attention to obtaining the equilibrium GB states considering the widespread presence of metastable GB states in polycrystalline materials. Equal emphasis should, at least, be placed on GB properties of metastable states. Zero or even negative vacancy formation energies are very likely to be observed under continuous vacancy loading according to the recent atomistic simulations conducted by Yu and Demkowicz^45^. They also stated that higher-energy GBs which have lower vacancy segregation energies are likely to be stronger vacancy sinks than lower-energy ones. In that sense, the high-angle GBs studies here might also have higher sink efficiencies than their low-angle counterparts. However, this hypothesis is also up for debate, since Jiang *et al*.^29^ demonstrated the contrary results that the larger stress fields of low-angle GBs lead to them having higher sink strength than high-angle GBs, which, in our case, is shown by the larger vacancy absorption length scales of low-angle GBs. Most recently, A. Vattre *et al*.^34^ also demonstrated the strong effect of the elastic stress fields in enhancing interface sink strength. More comprehensive models taking into account these important factors are called for to describe the complex defect evolution in materials upon irradiation.

In terms of the GB vacancy structures, our observation^46^ of possible vacancy delocalization in the dislocation core of the most GBs is consistent with the dislocation-based mechanism demonstrated by Yu and Demkowicz^45^, and the transmission electron microscopy observations by King and Smith^76^. We, however, have not detected such feature in some GBs such as ∑37(05-7) and ∑65(07-9) GBs, which have distinctively higher minimum vacancy segregation energies than its neighbours (see Fig. 5). However, it is reasonable to believe that the compact vacancies near the core are likely to delocalize at finite temperatures. A good test of the nature of the GB vacancies is to perform GB migration simulations (see Supplementary Figs S1 and S2 and the corresponding Supplementary Notes) at low temperatures and examine whether the GB vacancies can follow the migration of GBs. Since vacancies with compact nature are immobile at this temperature, they will inhibit GB migration and eventually be left behind by the migrating GBs. It is, nonetheless, still unclear to what extent will the materials be influenced by the compact vacancies. As we have demonstrated previously^46^, vacancies are both energetically and kinetically favourable to be absorbed by the GB dislocation cores and become delocalized. Therefore, vacancy delocalization might be an intrinsic feature in GBs.

It is currently well-accepted that equilibrium GBs can switch to different metastable states upon defect loading and absorption^33^,^39^,^45^ and the resulting metastable states can become better defect sinks^10^,^45^. In fact, the GB states can switch periodically upon continuous defect loading^33^,^45^, consistent with GBs being unsaturable or inexhaustible defect sinks. These observations also justify the self-healing mechanism proposed by Bai *et al*.^10^ that both interstitial emission and vacancy absorption can be activated at different temperature windows. Future works are underway to explore the influence of different GB character and states on the segregation energetics of impurities such as hydrogen and helium in bcc W. The obtained energetics and defect absorption length scales will provide useful inputs to the modelling and simulation of defect evolution and absorption at experimental time scales. For example, a rate-theory model on GB sink strength requires point defect formation energetics, absorption length scales, or defect migration barriers that can be readily obtained by atomistic simulations^29^,^34^. This will contribute to addressing the lifetime issue confronting the W-based plasma-facing components in future nuclear fusion devices.

## Conclusions

We have carried out molecular statics simulations to explore the energy landscapes of vacancy segregation to a range of equilibrium and metastable [100] symmetric tilt grain boundaries (STGBs) in bcc tungsten (W). The following conclusions can be drawn from this study.

1. For the equilibrium GBs, the magnitudes of minimum vacancy segregation energies vary from −2.61 to −0.76 eV, while those of mean vacancy segregation and absorption length scales range from −1.01 to −0.44 eV and from 3.18 to 12.41 Å, respectively, depending on the GB character.
2. Low-angle GBs have larger absorption length scales over their high-angle counterparts and the most energetically favourable vacancy sites are more often found at the first layer from the GB interface, while the repulsive sites are found in all GBs.
3. A qualitative analysis based on elasticity theory suggests that the first-order size effect and the second-order inhomogeneity effect dominate at the compressive and tensile region respectively.
4. There exist highly negative linear correlations between GB energy and mean vacancy segregation energy/vacancy absorption length scale for the equilibrium GBs.
5. The negative correlation between GB energy and mean vacancy segregation energy are applicable to GB metastable states as well. We further show that GB energy is affected by both the combination and the permutation of GB structural units.
6. The dislocation-based (delocalized) vacancy structure, which has been observed in many of the [100] STGBs studied here, may be an intrinsic feature of GBs, and it is of great significance in facilitating GB migration.
7. This study reveals the important effect of different GB character and states on vacancy segregation behavior and provides useful inputs for large-scale models, contributing to the possible application of polycrystalline W under irradiation in advanced nuclear fusion reactors.

## Methods

The interatomic potential for W-W interaction comes from earlier work of Ackland and Thetford^77^ and it was recently modified by Juslin and Wirth^78^ at the short range. This potential well reproduces the bulk properties and the vacancy formation energy of W as compared to experimental or DFT values^75^. Using the Large-scale Atomic/Molecular Massively Parallel Simulator (LAMMPS)^79^, we constructed the GB following the approach adopted in several previous studies^9^,^56^. First, based on the CSL theory, two differently oriented grains were brought together to obtain the bicrystal. Then, the two grains were translated rigidly with respect to each other, followed by an atom deletion process to avoid atomic overlap. The conjugate gradient (CG) algorithm was subsequently used for energy minimization. The GB energy *γ*_*GB*_ is calculated according to


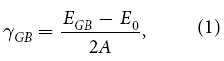


where *E*_GB_ and *E*_0_ represent the total energies of the supercell with and without a GB, respectively. *A* denotes the area of the GB interface. Since three-dimensional periodic boundary conditions were used, reasonably large cells (see Supplementary Table S1) were used to make sure that the interaction of the GBs and vacancies with their periodic images can be neglected. Tests on the possible size effect are presented in the Supplementary Fig. S7. The open software Ovito is used for structural visualization^80^.

Vacancies were systematically created by iteratively removing an atom within 18 Å of the STGBs. Only one vacancy was introduced into the cell at each simulation. After the CG energy minimization process, the formation energy can be obtained by


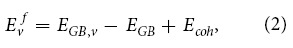


where *E*_*GB,v*_ represents the total energies of the GBs with a vacancy. *E*_*coh*_ is the cohesive energy per atom of a perfect bcc bulk lattice. Further, segregation energy is utilized to quantify the segregation tendency of a vacancy to the GBs, which can be simply expressed as


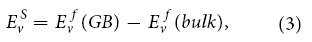


where 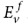 and 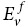 denote the vacancy formation energy in a perfect bcc bulk lattice and in a crystal with a GB, respectively. Thus, a negative segregation energy indicates attraction between the vacancy and the GB. Note that the local stress components were calculated using the viral stress tensor expression implemented in LAMMPS divided by the per-atom Voronoi volume.

## Additional Information

**How to cite this article**: Chen, N. *et al*. Energetics of vacancy segregation to [100] symmetric tilt grain boundaries in bcc tungsten. *Sci. Rep.* **6**, 36955; doi: 10.1038/srep36955 (2016).

**Publisher's note**: Springer Nature remains neutral with regard to jurisdictional claims in published maps and institutional affiliations.

## Supplementary Material

Supplementary Information
