# Supplementary material for: Energetics of vacancy segregation to [100] symmetric tilt grain boundaries in bcc tungsten
Source: Sci Rep. 2016 Nov 22;6:36955. doi: 10.1038/srep36955 (PMC5118800; doi:10.1038/srep36955)
Supplement: Supplementary Information [file srep36955-s1.pdf]

Supplementary Information

Energetics of vacancy segregation to [100] symmetric tilt grain boundaries in bcc tungsten

Nanjun Chen[1,3], Liang-Liang Niu[3,2], Ying Zhang[3], Xiaolin Shu[3], Hong-Bo Zhou[3], Shuo Jin[3], Guang Ran[1], Guang-Hong Lu[3] & Fei Gao[2]

[1]College of Energy, Xiamen University, Xiamen City, Fujian Province, 361102, China. [2]Department of Nuclear Engineering and Radiological Science, University of Michigan, Ann Arbor, MI 48109 USA. [3]Department of Physics, Beihang University, Beijing 100191, China. Correspondence and requests for materials should be addressed to Y.Z. (zhyi@buaa.edu.cn) or G.R. (gran@xmu.edu.cn) or F.G. (gaofeium@umich.edu).

Supplementary Notes

Using the same simulation methodology as described in our recent publication[1]. We have performed shear-coupled GB migration of two STGBs: the low-angle ∑85(07-6) GB and the high-angle ∑85(01-3). Fig. S1 shows the case of the low-angle ∑85(07-6) GB. Fig. S1a shows that all vacancies remain compact during the entire GB migration process and initial state is the global energy minimum. The increasing critical stress with the increasing number of vacancies suggests that vacancies keep their character and inhibit GB migration. All vacancies are left behind in the bulk. For Fig. S1b, the critical stress first deceases as the GB approaches the plane where the vacancies situate. Then these vacancies act as obstacles (shown by the higher critical stress) to the GB migration when the GB tries to break away from the vacancies. Interestingly, the critical stress for the 16-vacancy-loaded case is lower than that of the 8-vacancy-loaded one. This can be attributed to that several vacancies in the 16-vacancy-loaded case are absorbed by the GB dislocation core and become delocalized, which is also demonstrated by the noticeable energy drop. When it comes to Fig. S2 of the ∑13(01-5) GB, the decreasing critical stress with increasing number of vacancies indicates that vacancies are dislocation-based[2] and they facilitate GB migration. Consequently, no vacancies are left behind in the bulk. The results are in good agreement with previous literature[3].

Supplementary Figures

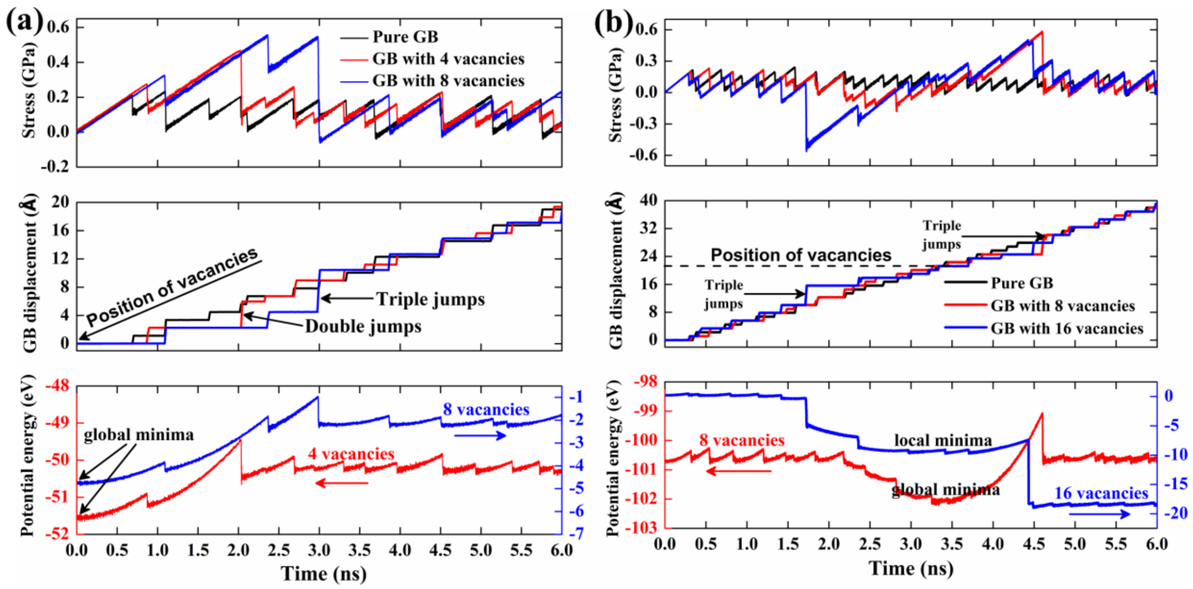

Figure S1. Shear-coupled migration of ∑85(07-6) GB loaded with different number of vacancies at 1 K. (a) Vacancies were created at the bulk-like region of the GB plane. (b) Vacancies were created at the (01-1) plane ~19 Å away from the GB plane. The simulation methodology of shear-coupled GB migration can be found in our recent publication[1].

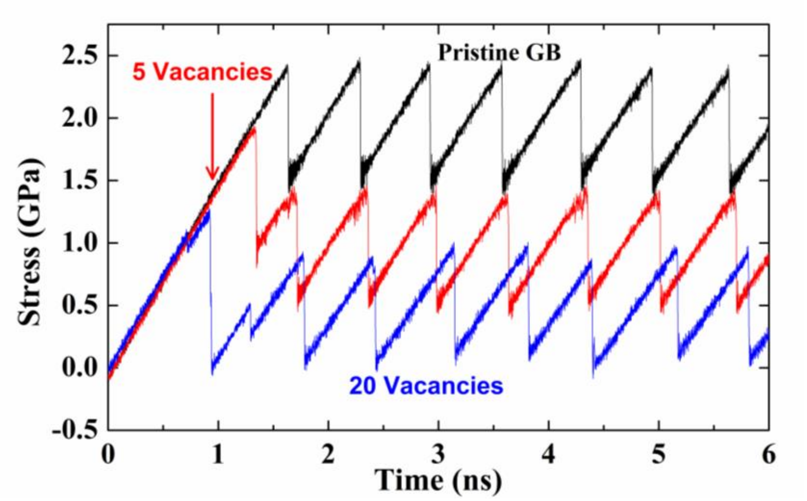

Figure S2. Shear-coupled migration of ∑13(01-5) GB loaded with different number of vacancies at 300 K. Vacancies are randomly placed at the GB plane.

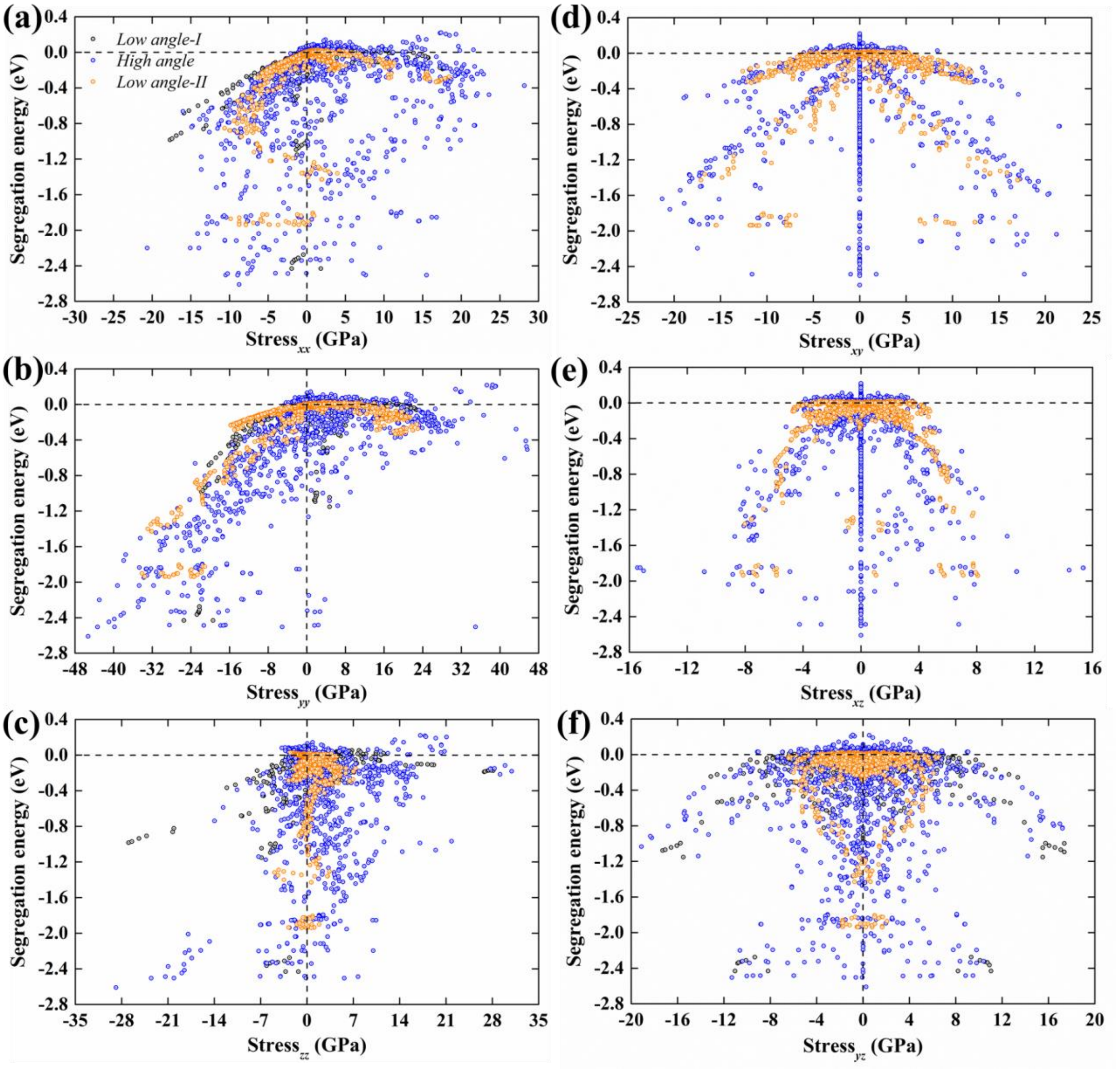

Figure S3. Dependence of vacancy segregation energy on six independent local normal (a) *xx*, (b) *yy*, (c) *zz* and shear (d) *xy*, (e) *xz*, (f) *yz* stress components for all the [100] STGBs. The dashed lines are guides for the eyes. It is demonstrated that both the normal and shear stress components contribute to the vacancy segregation energetics. For the normal stress components, we demonstrate that they affect the vacancy segregation energy asymmetrically, and the increasing compressive or tensile stress lowers the vacancy segregation energy with compressive stress playing a major role. For the effect of shear stress components, symmetric distributions can be observed, and the increasing absolute values of these components also contribute to lowering the vacancy segregation energy.

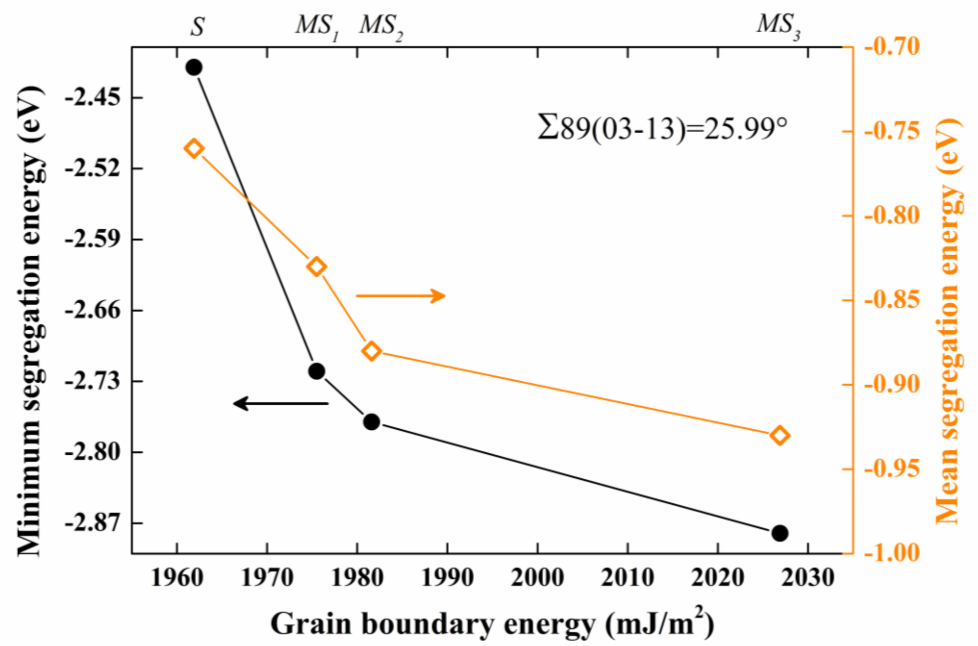

Figure S4. Dependence of minimum and mean vacancy segregation energy on GB energy for four different states of ∑89(03-13) GB. The lines are guide for the eyes. The representations of the GB states are the same as illustrated in the main text.

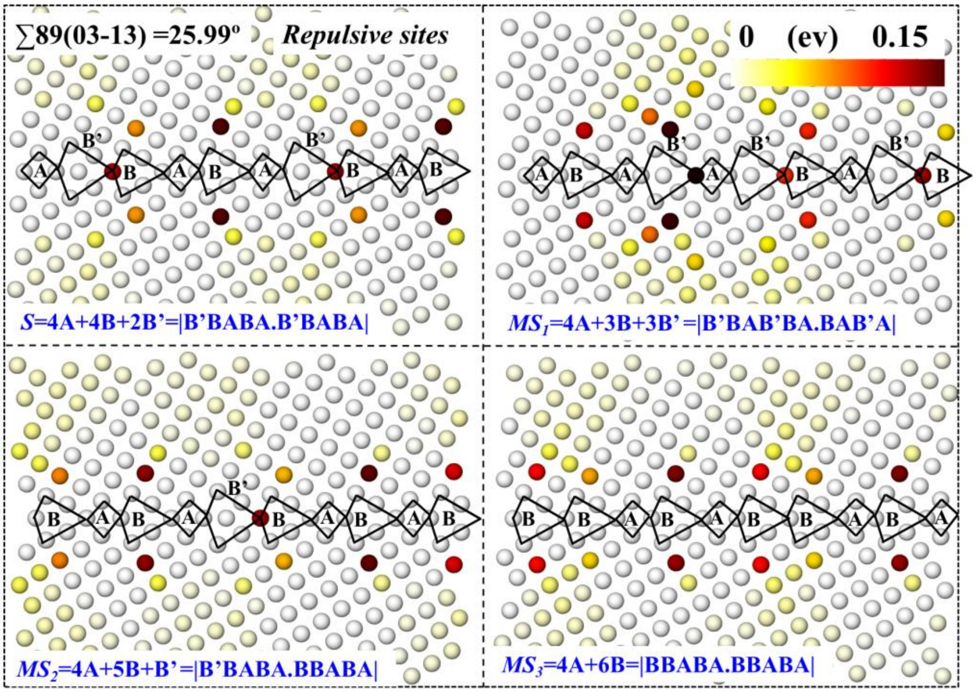

Figure S5. Vacancy segregation energy as a function of spatial position projected onto the (100) plane for ∑89(03-13) of different GB states. Only repulsive sites are shown.

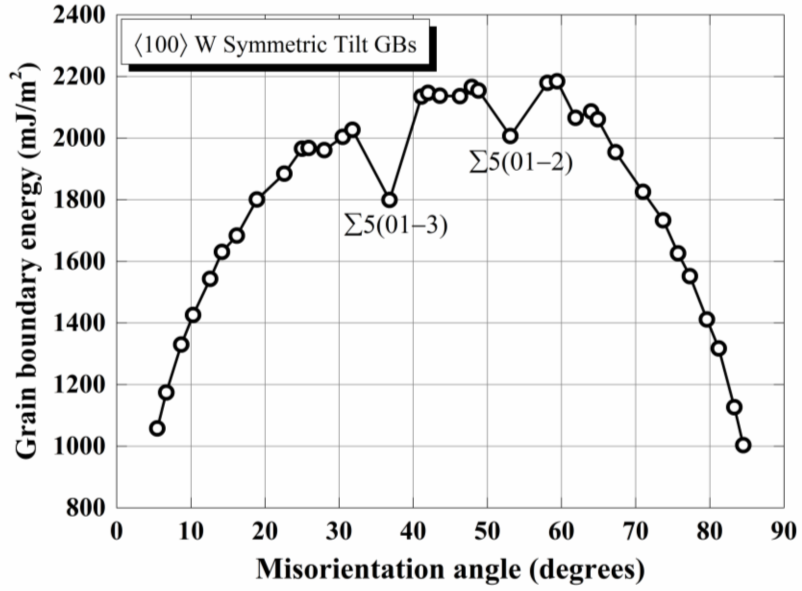

Figure S6. Grain boundary energy as a function of misorientation angle for the [100] STGBs in bcc W[1]. Notably, the qualitative correlation agrees well with that in atomistic simulations of [100] STGBs in bcc iron[4], tantalum[5] and molybdenum[6].

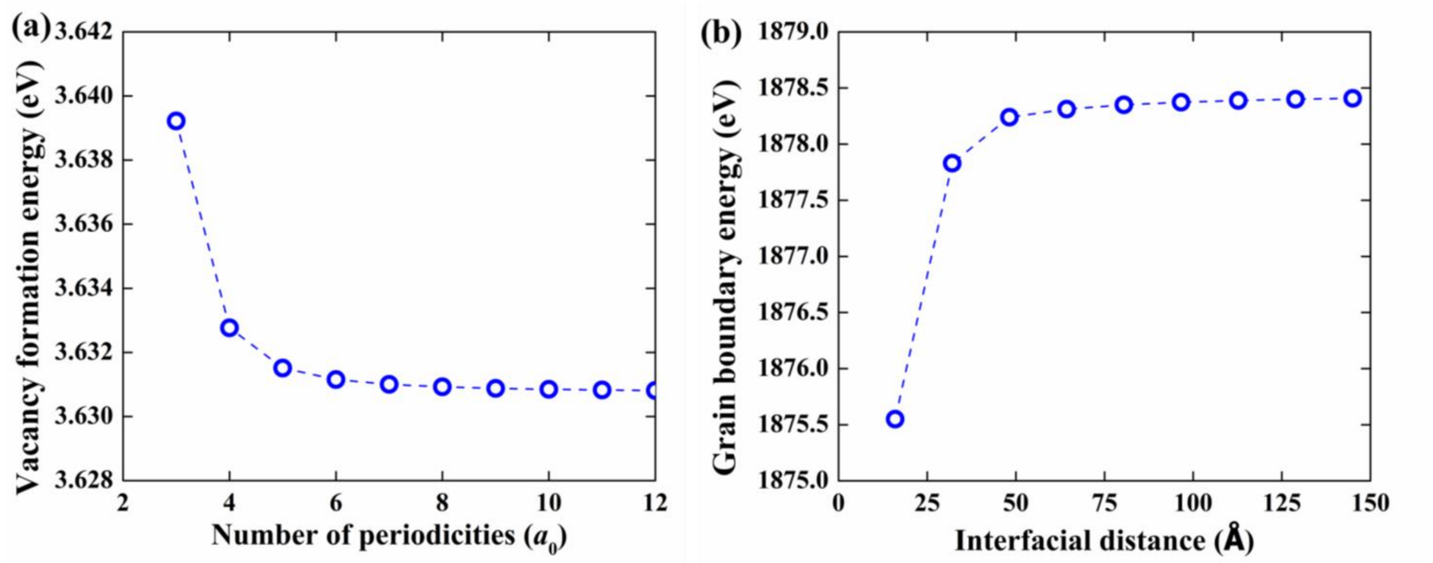

Figure S7. (a) Bulk vacancy formation energy as a function of the number of periodicities along each dimension. A choice of larger than $5a_0$ is good enough to minimize the size effect on vacancy formation. (b) Extra energies of the ∑13(01-5) GB as a function of the interfacial distance between the two neighboring periodic GBs. Grain boundary energy quickly reaches saturation at the short interfacial distance of ~50 Å. Since we use, in most cases, interfacial distances larger than 100 Å (see Supplementary Table S1), we thus believe that the size effect on GB energies can be neglected. The lines are guide for the eyes.

Table S1. Summary of characteristics and vacancy segregation properties of the [100] STGBs in W. Note that θ and γ are the GB misorientation angle and GB energy, respectively. x, y and z are the sizes of bicrystals. N is the number of atoms. $E_{s,min}$ and $E_{s,mean}$ are the minimum and mean vacancy segregation energy, respectively. L is the vacancy absorption length scale.

	Boundary	θ (°)	γ (mJ/m^2)	x (Å)	y (Å)	z (Å)	N	$E_{s,min}$ (eV)	$E_{s,mean}$ (eV)	L (Å)
Low angle-I	∑221(01-21)	5.45	1049.8	25.33	263.94	66.53	28048	-2.36	-0.44	8.22
	∑145(01-17)	6.73	1167.2	25.33	320.88	53.89	27616	-2.34	-0.45	8.19
	∑85(01-13)	8.80	1320.9	25.32	245.84	41.25	16192	-2.33	-0.51	6.65
	∑61(01-11)	10.39	1417.8	25.33	278.20	34.95	15520	-2.31	-0.56	6.60
	∑41(01-9)	12.68	1535.5	25.32	284.85	28.65	13024	-2.28	-0.61	6.53
	∑65(01-8)	14.25	1622.7	25.32	253.96	25.51	10336	-2.43	-0.64	5.84
High angle	∑25(01-7)	16.26	1676.9	25.32	267.23	44.74	19072	-2.19	-0.64	5.03
	∑37(01-6)	18.92	1793.9	25.32	268.55	38.49	16480	-2.34	-0.62	5.69
	∑13(01-5)	22.62	1878.4	25.32	257.72	32.25	13248	-1.95	-0.61	4.83
	∑85(02-9)	25.06	1959.4	25.32	289.04	29.17	13440	-2.45	-0.81	5.28
	∑89(03-13)	25.99	1961.9	25.32	337.03	42.21	22688	-2.42	-0.76	5.30
	∑17(01-4)	28.07	1953.8	25.31	260.02	26.09	10816	-2.35	-0.56	5.35
	∑65(03-11)	30.51	1997.7	25.31	287.62	36.08	16544	-2.48	-0.94	4.86
	∑53(02-7)	31.89	2020.0	25.31	275.32	23.04	10112	-2.50	-0.82	5.41
	∑5(01-3)	36.87	1796.0	25.30	259.12	30.02	12383	-0.76	-0.48	3.18
	∑73(03-8)	41.11	2127.4	25.30	267.62	27.03	11520	-2.49	-1.01	4.87
	∑97(05-13)	42.08	2139.4	25.31	264.17	44.08	18560	-2.61	-0.71	5.65
	∑29(02-5)	43.60	2130.5	25.31	272.19	34.08	14784	-2.22	-0.66	5.71
	∑29(03-7)	46.40	2128.0	25.31	237.84	24.10	9136	-2.37	-0.64	5.72
	∑97(04-9)	47.92	2157.8	25.31	246.10	31.17	12224	-2.50	-0.71	6.08
	∑73(05-11)	48.89	2148.1	25.32	305.50	38.24	18624	-2.16	-0.86	6.11
	∑5(01-2)	53.13	2000.0	25.31	251.22	28.30	11328	-1.42	-0.66	4.08
	∑53(05-9)	58.11	2170.5	25.32	257.32	32.57	13360	-2.20	-0.64	7.12
	∑65(04-7)	59.49	2176.9	25.31	252.77	25.51	10272	-2.12	-0.92	6.35
	∑17(03-5)	61.93	2060.5	25.31	329.82	36.89	19392	-1.41	-0.76	5.89
	∑89(05-8)	64.01	2080.3	25.31	297.74	29.84	14160	-2.04	-0.71	6.93
	∑85(07-11)	64.94	2053.9	25.31	246.47	41.23	16192	-1.85	-0.74	6.90
	∑13(02-3)	67.38	1945.6	25.31	225.47	34.20	12288	-1.51	-0.67	6.34
	∑37(05-7)	71.08	1817.0	25.31	271.42	27.21	11776	-1.44	-0.61	6.96
	∑25(03-4)	73.74	1724.5	25.31	189.20	31.62	9536	-1.84	-0.64	8.04

Low-angle-II	∑65(07-9)	75.75	1619.7	25.32	287.53	36.07	16544	-1.43	-0.54	9.14
	∑41(04-5)	77.32	1543.9	25.32	241.67	40.51	15616	-1.90	-0.62	9.14
	∑61(05-6)	79.61	1403.0	25.32	245.95	24.71	9696	-1.94	-0.55	9.14
	∑85(06-7)	81.20	1289.4	25.32	291.44	29.17	13568	-1.94	-0.52	9.66
	∑145(08-9)	83.27	1118.1	25.32	227.60	38.10	13840	-1.92	-0.53	11.33
	∑221(010-11)	84.55	994.1	25.32	187.13	47.04	14048	-1.90	-0.46	12.41

Supplementary References

1. Niu, L.-L. *et al.* Shear-coupled grain boundary migration assisted by unusual atomic shuffling. *Sci. Rep.* **6**, 23602 (2016).
2. Yu, W. S. & Demkowicz, M. J. Non-coherent Cu grain boundaries driven by continuous vacancy loading. *J. Mater. Sci.* **50**, 4047-4065 (2015).
3. Borovikov, V. *et al.* Influence of point defects on grain boundary mobility in bcc tungsten. *J. Phys.: Condens. Matter* **25**, 035402 (2013).
4. Tschopp, M. A. *et al.* Probing grain boundary sink strength at the nanoscale: Energetics and length scales of vacancy and interstitial absorption by grain boundaries in α-Fe. *Phys. Rev. B* **85**, 064108 (2012).
5. Hahn, E. N., Fensin, S. J., Germann, T. C. & Meyers, M. A. Symmetric tilt boundaries in body-centered cubic tantalum. *Scr. Mater.* **116**, 108-111 (2016).
6. Wolf, D. Correlation between the energy and structure of grain boundaries in b.c.c. metals. II. Symmetrical tilt boundaries. *Philos. Mag. A* **62**, 447-464 (1990).
